# Association of Hypomorphic P2X7 Receptor Genotype With Age

**DOI:** 10.3389/fnmol.2020.00008

**Published:** 2020-02-05

**Authors:** Juana Maria Sanz, Simonetta Falzoni, Mario Luca Morieri, Angelina Passaro, Giovanni Zuliani, Francesco Di Virgilio

**Affiliations:** ^1^Section of Internal and Cardiorespiratory Medicine, Department of Medical Sciences, University of Ferrara, Ferrara, Italy; ^2^Section of Pathology, Oncology and Experimental Biology, Department of Morphology, Surgery and Experimental Medicine, University of Ferrara, Ferrara, Italy

**Keywords:** aging, P2X7, inflammation, polymorphisms, neurodegeneration

## Abstract

One of the main risk factors for brain diseases is aging. Recent studies have shown that aging is a progressive degenerative process associated with chronic low-level inflammation. The ATP-gated P2X7 receptor (P2X7R) plays an important role in inflammation and has been associated with different brain (e.g., Alzheimer’s and Parkinson’s) or other age-related (osteoporosis, arthritis, cancer) diseases. Several single nucleotide polymorphisms (SNPs) in the *P2RX7* gene have been identified, including the loss-of-function 1513A>C and 1405A>G SNPs, and the gain-of-function 489C>T and 1068G>A SNPs. We carried out a literature analysis to verify an association between* P2RX7* SNPs’ frequency and age. In 34 worldwide eligible studies (11.858 subjects) no correlation between 1513CC genotype frequency and age emerged. On the contrary, analysis of European Caucasian cohorts (7.241 subjects) showed a significant increase in 1513CC frequency with age (*P* = 0.027). In agreement with these findings, analysis of two publicly available datasets, including USA Caucasian cohorts, unveiled an increased frequency of 1513CC and 489CC genotypes with age (*P* = 0.0055 and *P* = 0.0019, respectively). Thus, hypomorphic *P2RX7* genotypes may be positively selected with age in European and North American Caucasian populations. We hypothesize that Caucasian individuals bearing an anti-inflammatory P2X7R phenotype and living in high-income countries may have a longer life expectancy.

## Introduction

Prevalence of central nervous system (CNS) diseases increases with age, either directly, by a time-dependent accumulation and aggregation of abnormal proteins, e.g., in Alzheimer’s, Parkinson’s, and Huntington’s disease, or indirectly, due to the increase in age-related changes that foster the onset and/or progression of brain diseases. For example, stroke is associated with a high risk of seizures and epilepsy, while type 2 diabetes and atherosclerosis are a risk factor for Alzheimer’s and cerebrovascular disease (Lénárt et al., 2016; Beghi and Giussani, 2018; Hou et al., 2019).

On the other hand, inflammation is a well-recognized pathogenic factor in age-associated disorders, neurological disorders included. A role for chronic, low level, systemic inflammation is hypothesized in psychiatric conditions, epilepsy, cerebrovascular diseases, dementia and neurodegeneration (Vezzani et al., 2011; Najjar et al., 2013; Lénárt et al., 2016; Guzman-Martinez et al., 2019; Ignácio et al., 2019).

The P2X7 receptor (P2X7R) is an ATP-gated cation-selective channel involved in inflammation and host defense. P2X7R activation promotes the release of several pro-inflammatory factors, both in the CNS and in peripheral tissues, and is understood to participate in the pathogenesis of several neurodegenerative diseases such as multiple sclerosis, Alzheimer’s, Parkinson’s, and Huntington’s disease (Savio et al., 2018; Kanellopoulos and Delarasse, 2019). The P2X7R is also involved in the pathogenesis of age-related pathologies such as cancer, osteoporosis, diabetes and arthritis (Tao et al., 2013; Kvist et al., 2014; Sperlágh and Illes, 2014; Novak and Solini, 2018; Adinolfi et al., 2019). The strong pro-inflammatory activity of the P2X7R depends on the ability of this receptor to trigger the generation of reactive oxygen species and release of cytokines and metalloproteases. Some of these responses are mediated through the stimulation of the NLRP3 inflammasome and of caspase-1. P2X7R activation may have opposite effects on cell growth; low level, tonic stimulation promotes cell proliferation, while sustained stimulation triggers cell death by necrosis or apoptosis (Di Virgilio, 2013). P2X7R-dependent cytotoxicity can be exploited for intracellular pathogen killing (Adinolfi et al., 2018).

*P2RX7* is a highly polymorphic gene located on chromosome 12q24.31. The most studied *P2RX7* single nucleotide polymorphism (SNP) is the 1513A>C. Homozygous subjects carrying the 1513CC genotype show a non-functional P2X7R pore and a reduced ability to activate inflammation compared to wild-type subjects bearing the AA genotype (Wesselius et al., 2012). The possible association of the 1513CC *P2RX7* genotype with different inflammatory conditions is attracting increasing interest (Di Virgilio et al., 2017). Other important *P2RX7* SNPs are the loss-of-function 1405A>G, and the gain-of-function 489C>T and 1068G>A (Sluyter and Stokes, 2011; Caseley et al., 2014).

In the present study, we tested the hypothesis of an association between age and frequency among polymorphic P2X7 receptor genotypes. To this aim, we carried out a revision of the relevant literature and the analysis of two dbGaP (database Genotype and Phenotype) datasets.

## Materials and Methods

### Publication Search Strategy

A Medline literature search using the keywords rs3751143, 1513A>C, or E496A, which identify the *P2RX7* SNP of interest, allowed a partial retrieval of all pertinent studies. Therefore, the search was extended to the keyword mesh “(P2X7 or P2X7R or P2RX7) and (polymorphism or polymorphisms).” In July 2016, this search produced 178 hits, from which 79 articles analyzing the frequency of 1513A>C *P2RX7* SNP were selected. Forty seven studies were excluded because: (a) two were based on small cohorts (16 and 46, respectively); (b) seven analyzed only diseased subjects, with no cohorts comprising healthy controls; (c) four reported data from already published control cohorts; (d) one article was not found; and (e) the remaining 33 studies did not specify the mean or median age and/or CC frequency of the control cohorts. Thirty-two studies involving a total 34 cohorts (Zhang et al., 2003; Fernando et al., 2007 articles describe two different control cohorts) with 11,858 subjects, were thus identified. With the exception of the study by Sambasivan (Sambasivan et al., 2010), genotype distribution in all control cohorts was in Hardy–Weinberg equilibrium (HWE).

### dbGaP Analysis

We received NIH approval to analyze two datasets comprising *P2RX7* SNPs in Caucasian control cohorts which report the age of enrolled subjects:

1. HGVST1 (Human Genoma Variation ST1); Study of prostate cancer; dbGaP Study Accession, phs000207.v1.p1. Dataset Name: CGEMS (The Cancer Genetic Markers of Susceptibility) Prostate_Data; Dataset Accession, pht001105.v1.p1); NIH approval, [#47650-2] [#47650-4]. It is a nested case-control study to identify SNP associated to augmented prostate cancer susceptibility. Control cohort include 1,101 men with European ancestry selected from The Prostate, Lung, Colon and Ovarian (PLCO) Cancer Screening Trial from USA (Yeager et al., 2007).
2. HGVST6; Study of Parkinson’s Disease; dbGaP Study Accession, phs000089.v3.p2; Dataset Name cde_ctl. Dataset Accession, pht000177.v3.p2; NIH approval accession: [#47649-3]. This case-control study analyzed genetic variants that may increase risk of Parkinson’s disease in the collection of North American Caucasians with Parkinson’s disease, as well as neurologically normal controls from the sample population which are banked in the National Institute of Neurological Disorders and Stroke (NINDS Repository) collection (Fung et al., 2006). The control cohort is composed of 802 Caucasian subjects, about 60% are women, and more than 95% of the subjects originate from the USA. Each participant underwent a detailed medical history interview and had no family history of Alzheimer’s disease, amyotrophic lateral sclerosis, ataxia, autism, bipolar disorder, brain aneurysm, dementia, dystonia, or Parkinson’s disease.

In these control cohorts, we have searched 16 characterized *P2RX7* SNPs (Sluyter and Stokes, 2011) out of more than 300,000 SNPs reported in the databases, but only four polymorphisms were identified. The main published features of these *P2RX7* SNP polymorphisms are shown in Table 1.

**Table 1:** Principal published characteristics of *P2RX7* SNP polymorphisms.

	Base change	Amino acid change	Effect on function	Minor allele frequency
rs2230912	1405A > G	Gln460Arg	Partial loss	0.17
rs3751143	1513A > C	Glu496Ala	Loss	0.175
rs1718119	1068G > A	Ala348Thr	Gain	0.400
rs208294	489C > T	His155Tyr	Gain	0.439

In the HGVST6 dataset, individual subject age was specified, while in the HGVST1 dataset only decade age was reported, therefore to make data from both datasets homogenous, subjects from the HGVST6 study were re-comprised in the same age decade sub-cohorts as the HGVST1 study, as follows: decade # 3, age range 15–29 (number of subjects, 51); decade # 4, age range 30–39 (number of subjects, 77); decade # 5, age range 40–49 (number of subjects, 99); decade # 6, age range 50–59 (number of subjects, 280); decade # 7, age range 60–69 (number of subjects, 821); decade # 8, age range 70–79 (number of subjects, 507); decade # 9, age range 80–94 (number of subjects, 68). Age decade # 3 was not included in the analysis due to its small number of subjects and because, according to the USA Center for Diseases Control and Prevention (CDC) and the Word Health Association (WHO), the three main causes of death between 15 and 30 years are unintentional injury, suicide and homicide (more than 70% of total deaths), none of which are associated with inflammation^1^,^2^. All four *P2RX7* SNPs analyzed were in the HWE across all age decades, with the exception of the gain-of-function rs208294 SNP in age decade # 5 (*p* = 0.037).

### Statistical Analysis

Data on the rs2230912, rs3751143, rs1718119, rs208294 genotypes, and the age of the subjects enrolled in the two dbGaP datasets, were extracted using SAS 9.4 (SAS Institute, Cary, NC, USA), and analyzed by correlation analysis using the GraphPad InStat 3 software (Graphpad Software, San Diego, CA, USA). The KS normality test (Kolmogorov–Smirnov tests with Dallal–Wilkinson–Liliefor *P*-value) was applied, and the Pearson correlation coefficient was calculated. Statistical significance was assumed as *p* < 0.05 for the initial rs375114 studies. When we subsequently analyzed data from the dbGaP and tested three other P2X7 SNPs as a secondary objective (considering eight test hypotheses and according with the Bonferroni correction), statistical significance was reduced to *p* < 0.00625.

## Results and Discussion

The 1513A>C *P2RX7* SNP was initially identified in monocytes from a healthy subject with a nonfunctional P2X7 (Gu et al., 2001). Later it was associated with many different diseases, including tuberculosis, Crohn’s disease, rheumatoid arthritis, and psychiatric disorders (Sluyter and Stokes, 2011). Our previous studies showed a higher frequency of the 1513CC *P2RX7* genotype in aged compared with young cohorts, but the sample size was rather small (Cabrini et al., 2005; Dardano et al., 2009; Sanz et al., 2014). Thus, we decided to perform a wide range literature search in PubMed, using the queries “(P2X7 or P2X7R or P2RX7) and (polymorphisms or polymorphism).” Source and data extracted from 34 healthy cohorts specifying mean or median age (29 and 5 cohorts, respectively) and CC % frequency, analyzed across 32 articles, are shown in Table 2.

**Table 2:** Age and 1513CC *P2RX7* genotype frequency in different population cohorts.

Cohort origin	Mean/Median age (years) ± SD (age range)	Genotype 1513CC Freq. (%)	Subjects number	References
China	5.9 ± 4.0 (0.25–16)	11.5	384	Xiao et al. (2009)
Turkey	7.8 ± 4.9	2.6	192	Tekin et al. (2010)
United Kingdom	29 (10–49)	3.0	235	Zhang et al. (2003)
Gambia	30.3 ± 7.5	1.3	297	Li et al. (2002)
Russia	32.2 ± 12.0 (21–71)	2.4	126	Mokrousov et al. (2008)
Peru	32.6 ± 9.4	3.3	513	Taype et al. (2010)
Brazil	32.8 ± 16.5	3.0	263	de Salles et al. (2017)
Oman	35 ± 7	8.2	158	Al-Shukaili et al. (2011)
Tunisia	35 (24–55)	4.0	150	Ben-Selma et al. (2011)
India	35.6 ± 13.3	8.0	100	Sambasivan et al. (2010)
Brazil	35.8 ± 12.0	5	288	Souza de Lima et al. (2016)
Turkey	36.3 ± 19.7 (2–86)	3	120	Somuk et al. (2016)
India	36.4 ± 14.9	2.8	392	Singla et al. (2012)
China	37.2 ± 16.6 (9–80)	6.2	532	Chen et al. (2013)
Australia	37.8 ± 13.0	3.9	102	Fernando et al. (2007)
Turkey	39.3 ± 13.8	13.1	160	Özdemir et al. (2014)
Germany	39.8 ± 11.4	4.6	461	Erhardt et al. (2007)
Korea	40.7	4.0	150	Lee et al. (2007)
Iran	43	1.0	100	Shamsi et al. (2016)
Italy	44.1 ± 12.8	2.0	100	Dardano et al. (2009)
Denmark	44.6 ± 12.2 (21–88)	2.1	808	Hansen et al. (2008)
Australia	46.1 ± 8.9	4.2	167	Fernando et al. (2007)
Italy	46.7 ± 11.1	3.8	131	Ghiadoni et al. (2013)
China	47.0 ± 14.5	10.4	87	Wu et al. (2015)
Denmark	50.7 (45–58)	2.7	1,764	Ohlendorff et al. (2007)
India	55.2 (40–80)	1.7	177	Sharma et al. (2010)
United Kingdom	58 ± 12	4.0	428	Sellick et al. (2004)
United Kingdom	*58* (50–90)	3.5	113	Zhang et al. (2003)
Sweden	61 (49–75)	5.0	200	Thunberg et al. (2002)
Germany	62	5.2	97	Nückel et al. (2004)
Sweden	63 ± 6.5	3.2	2,404	Gidlöf et al. (2012)
Denmark	65.3 ± 8.2	3.5	226	Husted et al. (2013)
China	71.8 ± 6.1	2.8	285	Liu et al. (2013)
Italy	73 ± 5.6 (65–93)	7.4	148	Sanz et al. (2014)

*Age is specified as found in the original reference: Mean ± SD or Median (age range)*.

Cohorts were further subdivided into two groups, European (13 articles comprising 14 cohorts) and non-European (19 articles comprising 20 cohorts). Ethnic origin of the cohorts was specified in five European studies (Caucasian origin) and in six non-European studies (non-Caucasian origin). In the remaining cohorts, ethnic origin was not specified, and healthy control subjects were local volunteers. Thus, it was assumed that the majority of the participants belonged to the prevalent ethnicity in the given country.

**Figure 1:**
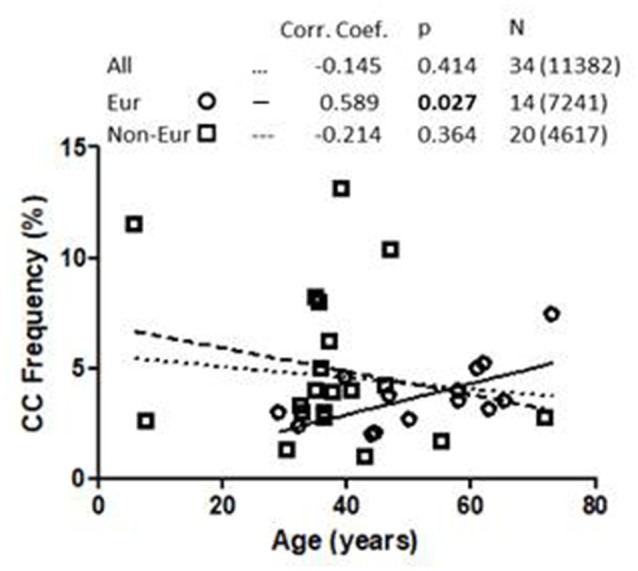
Association of 1513CC P2X7 genotype frequency with the age in All, European and Non-European cohorts. Legend. Corr. Coef., Pearson correlation coefficient. N: cohort number (total number of subjects).

Linear regression analysis of the association between 1513CC *P2RX7* frequency and age from all cohorts, both European and non-European, is shown in Figure 1. Analysis of pooled data from all cohorts showed no correlation between 1513CC *P2RX7* frequency and age. Likewise, no correlation between 1513CC *P2RX7* frequency and age was observed in non-European cohorts. On the contrary, subgroup analysis of European cohorts showed a significant correlation of 1513CC *P2RX7* frequency with age (*p* = 0.027). Separate analysis of European countries with three or more cohorts: Italy (Dardano et al., 2009; Ghiadoni et al., 2013; Sanz et al., 2014), Denmark (Ohlendorff et al., 2007; Hansen et al., 2008; Husted et al., 2013), and the United Kingdom (Starczynski et al., 2003; Zhang et al., 2003; Sellick et al., 2004), numbering three, three, and four cohorts respectively, showed a trend of increase in 1513CC *P2RX7* frequency with age.

To further validate data derived from the literature, we analyzed 1513CC frequency in 1903 Caucasian control subjects included in the HGVST1 and HGVST6 studies. As a secondary objective, three other *P2RX7* SNPs (namely, the 489C>T loss-of-function, the 1068G>A and 1405A>G gain-of-function SNPs) were also included in this analysis (Table 3).

**Table 3:** *P2XR7* genotype frequency in HGVST1 and HGVST6 dataset.

	Hypomorphic P2X7R	Intermediate P2X7R	Hypermorphic P2X7R	Minor allele frequency	Number of subjects*
1405A > G	GG (%) 44 (2.3%)	AG (%) 540 (28.4%)	AA (%) 1,318 (69.3%)	0.165	1,902
1513A > C	CC (%) 61 (3.3%)	AC (%) 602 (32.5%)	AA (%) 1,192 (64.3%)	0.195	1,855
1068G > A	GG (%) 701 (37%)	AG (%) 932 (49.2%)	AA (%) 263 (13.9%)	0.384	1,896
489C > T	CC (%) 543 (28.5%)	CT (%) 993 (52.2%)	TT (%) 367 (19.3%)	0.454	1,903

* Number subject varies because for some individuals some genotypes are non-specified in the datasets.

**Figure 2:**
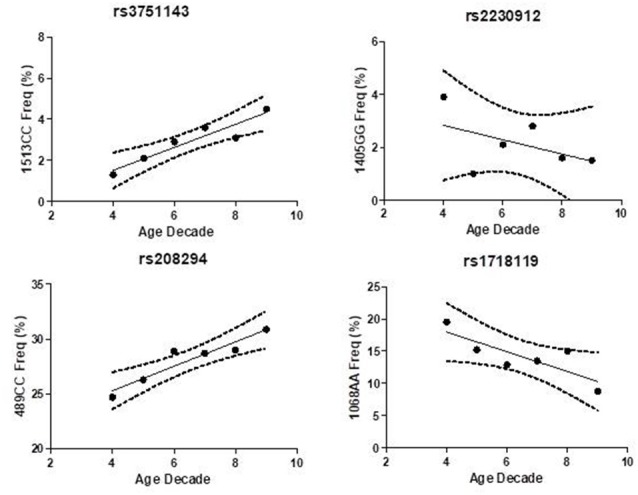
Association of P2X7 genotypes frequency with age decade in Caucasian control cohort.

Hypomorphic and hypermorphic P2X7R genotype frequency of all SNPs at different age decades was analyzed to verify either a frequency increase in hypomorphic receptor or a frequency decrease of hypermorphic receptor with age (Table 4). A statistically-significant association between the increase in hypomorphic 1513CC and 489CC genotype frequency with age was found, and a reduction in the hypermorphic 1068AA genotype frequency with age was observed, but statistical significance was not reached. Instead, 1405A>G SNP frequency was independent of age (Figure 2). It is worth mentioning that a decreased frequency of a gain-of-function SNP (308GG) with age has also been reported for the potent pro-inflammatory cytokine TNFα (Cardelli et al., 2008).

**Table 4:** Correlation analysis of *P2XR7* genotype frequency with age decade.

SNP	Hypomorphic P2X7R			Hypermorphic P2X7R		
	Genotype	Corr. Coef.	*p*	Genotype	Corr. Coef.	*p*
1405A > G	1405GG	−0.4826	0.3323	1405AA	−0.00817	0.9877
1513A > C	1513CC	0.9390	0.0055^#^	1513AA	−0.4904	0.3234
1068G > A	1068GG	0.3983	0.4342	1068AA	−0.8184	0.0465
489C > T	489CC	0.9359	0.0019^#^	489TT	−0.08178	0.8616

*^#^*p* < 0.00625*.

Differences in 1513CC frequency between European/USA and non-European/non-USA cohorts may be due to ethnic background, as previously reported for immune system-related genes, *P2RX7* included (Lindenau et al., 2013). Also, as suggested by Fuller et al. (2009), environmental factors and prevalent diseases might also cause an allelic selection of *P2RX7* SNPs. Environmental factors, such as hygienic conditions, climate, and food availability, which are extremely variable in different areas of world, have a strong influence on disease prevalence and life expectancy. In low-income countries, nearly 40% of deaths occur in childhood (0 to 15-years age range), while only 20% occur among aged people (70 years and older). In these countries, morbidity and mortality are mainly due to infectious diseases (e.g., lower respiratory tract infections, HIV/AIDS, diarrheal diseases, malaria, and tuberculosis) that collectively account for almost one third of all deaths (World Health Organization data). The P2X7R has been reported to have a protective action against some common infective pathogens, such as Plasmodium, Mycobacterium, and Chlamydia. In high-income countries, however, 70% of deaths occur among people aged 70 years and older, the main causes being chronic diseases where inflammation plays an important and detrimental role, including cardiovascular disease, cancer, dementia, chronic obstructive pulmonary disease, and diabetes (World Health Organization data). Under these conditions, reduced activity of a potent pro-inflammatory receptor such as the P2X7R may turn out to be beneficial. Finally, a limitation of our study is the reduced sample size. Further replication studies are needed to test our hypothesis.

## Conclusion

Based on these results, we hypothesize that in Caucasian elderly populations from high-income countries, where a hypofunctional P2X7R might afford protection against prevalent chronic inflammatory diseases, hypomorphic *P2RX7* alleles may be positively selected with age. This hypothesis suggests that the P2X7R might be a therapeutic target to alleviate inflammatory brain disorders and others age-related diseases.

## Data Availability Statement

The datasets analyzed for this study can be found in two public available datasets: HGVST1 and HGVST6.

## Author Contributions

JS: study design, bibliographic research, statistical analysis, writing and discussion of the manuscript. SF: critical reading of the manuscript. MLM: dbGaP statistical analysis and critical reading of the manuscript. GZ and AP: critical reading and discussion of the manuscript. FDV: writing, critical reading and discussion of the manuscript.

## Conflict of Interest

FDV is a member of the Scientific Advisory Board of Biosceptre Limited, a UK-based biotech Company involved in the development of P2X7R-targeted therapeutics.

The remaining authors declare that the research was conducted in the absence of any commercial or financial relationships that could be construed as a potential conflict of interest.

## Footnotes

^1^https://www.cdc.gov/injury/wisqars/pdf/leading_causesofdeathbyagegroup2015-a.pdf

^2^http://www.who.int/healthinfo/global_burdendisease/estimates/en/index1.html

## References

[B1] Adinolfi E. De Marchi E. Orioli E. Pegoraro A. Di Virgilio F. (2019). Role of the P2X7 receptor in tumor-associated inflammation. Curr. Opin. Pharmacol. 47, 59–64. 10.1016/j.coph.2019.02.012 30921559

[B2] Adinolfi E. Giuliani A. L. De Marchi E. Pegoraro A. Orioli E. Di Virgilio F. (2018). The P2X7 receptor: a main player in inflammation. Biochem. Pharmacol. 151, 234–244. 10.1016/j.bcp.2017.12.021 29288626

[B3] Al-Shukaili A. Al-Kaabi J. Hassan B. Al-Araimi T. Al-Tobi M. Al-Kindi M.. (2011). P2X7 receptor gene polymorphism analysis in rheumatoid arthritis. Int. J. Immunogenet. 38, 389–396. 10.1111/j.1744-313X.2011.01019.x 21645266

[B4] Beghi E. Giussani G. (2018). Aging and the epidemiology of epilepsy. Neuroepidemiology 51, 216–223. 10.1159/000493484 30253417

[B5] Ben-Selma W. Ben-Kahla I. Boukadida J. Harizi H. (2011). Contribution of the P2X71513A/C loss-of-function polymorphism to extrapulmonary tuberculosis susceptibility in Tunisian populations. FEMS Immunol. Med. Microbiol. 63, 65–72. 10.1111/j.1574-695x.2011.00824.x 21635566

[B6] Cabrini G. Falzoni S. Forchap S. L. Pellegatti P. Balboni A. Agostini P.. (2005). A His-155 to tyr polymorphism confers gain-of-function to the human P2X_7_ receptor of human leukemic lymphocytes . J. Immunol. 175, 82–89. 10.4049/jimmunol.175.1.82 15972634

[B7] Cardelli M. Cavallone L. Marchegiani F. Oliveri F. Dato S. Montesanto A.. (2008). A genetic-demographic approach reveals male-specific association between survival and tumor necrosis factor (A/G)-308 polymorphism. Journals Gerontol. A Biol. Sci. Med. Sci. 63, 454–460. 10.1093/gerona/63.5.454 18511747

[B8] Caseley E. A. Muench S. P. Roger S. Mao H. J. Baldwin S. A. Jiang L. H. (2014). Non-synonymous single nucleotide polymorphisms in the P2X receptor genes: association with diseases, impact on receptor functions and potential use as diagnosis biomarkers. Int. J. Mol. Sci. 15, 13344–13371. 10.3390/ijms150813344 25079442 PMC4159798

[B9] Chen G. M. Feng C. C. Ye Q. L. Tao J. H. Li R. Peng H.. (2013). Association of *P2X7R* gene polymorphisms with systemic lupus erythematosus in a Chinese population. Mutagenesis 28, 351–355. 10.1093/mutage/get007 23435013

[B10] Dardano A. Falzoni S. Caraccio N. Polini A. Tognini S. Solini A.. (2009). 1513A >C polymorphism in the P2X_7_ receptor gene in patients with papillary thyroid cancer: correlation with histological variants and clinical parameters. J. Clin. Endocrinol. Metab. 94, 695–698. 10.1210/jc.2008-1322 19017759

[B38] de Salles É. M. de Menezes M. N. Siqueira R. Borges da Silva H. Amaral E. P. Castillo-Méndez S. I.. (2017). P2X7 receptor drives Th1 cell differentiation and controls the follicular helper T cell population to protect against *Plasmodium chabaudi* malaria. PLoS Pathog. 13:e1006595. 10.1371/journal.ppat.1006595 28859168 PMC5597262

[B11] Di Virgilio F. (2013). The therapeutic potential of modifying inflammasomes and NOD-like receptors. Pharmacol. Rev. 65, 872–905. 10.1124/pr.112.006171 23592611

[B12] Di Virgilio F. Dal Ben D. Sarti A. C. Giuliani A. L. Falzoni S. (2017). The P2X7 receptor in infection and inflammation. Immunity 47, 15–31. 10.1016/j.immuni.2017.06.020 28723547

[B13] Erhardt A. Lucae S. Unschuld P. G. Ising M. Kern N. Salyakina D.. (2007). Association of polymorphisms in P2RX7 and CaMKKb with anxiety disorders. J. Affect. Disord. 101, 159–168. 10.1016/j.jad.2006.11.016 17197037

[B14] Fernando S. L. Saunders B. M. Sluyter R. Skarratt K. K. Goldberg H. Marks G. B.. (2007). A polymorphism in the P2X7 gene increases susceptibility to extrapulmonary tuberculosis. Am. J. Respir. Crit. Care Med. 175, 360–366. 10.1164/rccm.200607-970OC 17095747

[B15] Fuller S. J. Stokes L. Skarratt K. K. Gu B. J. Wiley J. S. (2009). Genetics of the P2X7 receptor and human disease. Purinergic Signal. 5, 257–262. 10.1007/s11302-009-9136-4 19319666 PMC2686826

[B16] Fung H. C. Scholz S. Matarin M. Simón-Sánchez J. Hernandez D. Britton A.. (2006). Genome-wide genotyping in Parkinson’s disease and neurologically normal controls: first stage analysis and public release of data. Lancet Neurol. 5, 911–916. 10.1016/S1474-4422(06)70578-6 17052657

[B17] Ghiadoni L. Rossi C. Duranti E. Santini E. Bruno R. M. Salvati A.. (2013). P2X7 receptor polymorphisms do not influence endothelial function and vascular tone in neo-diagnosed, treatment-naive essential hypertensive patients. J. Hypertens. 31, 2362–2369. 10.1097/hjh.0b013e3283653ff5 24220590

[B18] Gidlöf O. Smith J. G. Melander O. Lövkvist H. Hedblad B. Engström G.. (2012). A common missense variant in the ATP receptor P2X7 is associated with reduced risk of cardiovascular events. PLoS One 7:e37491. 10.1371/journal.pone.0037491 22662160 PMC3360776

[B19] Gu B. J. Zhang W. Worthington R. A. Sluyter R. Dao-Ung P. Petrou S.. (2001). A Glu-496 to ala polymorphism leads to loss of function of the human P2X_7_ receptor. J. Biol. Chem. 276, 11135–11142. 10.1074/jbc.M010353200 11150303

[B20] Guzman-Martinez L. Maccioni R. B. Andrade V. Navarrete L. P. Pastor M. G. Ramos-Escobar N. (2019). Neuroinflammation as a common feature of neurodegenerative disorders. Front. Pharmacol. 10:1008. 10.3389/fphar.2019.01008 31572186 PMC6751310

[B21] Hansen T. Jakobsen K. D. Fenger M. Nielsen J. Krane K. Fink-Jensen A.. (2008). Variation in the purinergic P2RX_7_ receptor gene and schizophrenia. Schizophr. Res. 104, 146–152. 10.1016/j.schres.2008.05.026 18614336

[B22] Hou Y. Dan X. Babbar M. Wei Y. Hasselbalch S. G. Croteau D. L.. (2019). Ageing as a risk factor for neurodegenerative disease. Nat. Rev. Neurol. 15, 565–581. 10.1038/s41582-019-0244-7 31501588

[B23] Husted L. B. Harsløf T. Stenkjær L. Carstens M. Jørgensen N. R. Langdahl B. L. (2013). Functional polymorphisms in the P2X_7_ receptor gene are associated with osteoporosis. Osteoporos. Int. 24, 949–959. 10.1007/s00198-012-2035-5 22707062

[B24] Ignácio Z. M. da Silva R. S. Plissari M. E. Quevedo J. Réus G. Z. (2019). Physical exercise and neuroinflammation in major depressive disorder. Mol. Neurobiol. 56, 8323–8335. 10.1007/s12035-019-01670-1 31228000

[B25] Kanellopoulos J. M. Delarasse C. (2019). Pleiotropic roles of P2X7 in the central nervous system. Front. Cell. Neurosci. 13:401. 10.3389/fncel.2019.00401 31551714 PMC6738027

[B26] Kvist T. M. Schwarz P. Jørgensen N. R. (2014). The P2X7 receptor: a key player in immune-mediated bone loss? Scientific World Journal 2014:954530. 10.1155/2014/954530 24574936 PMC3915485

[B27] Lee K. Park S. S. Kim I. Kim J. H. Ra E. K. Yoon S.. (2007). P2X_7_ receptor polymorphism and clinical outcomes in HLA-matched sibling allogeneic hematopoietic stem cell transplantation. Haematologica 92, 651–657. 10.3324/haematol.10810 17488689

[B28] Lénárt N. Brough D. Dénes Á. (2016). Inflammasomes link vascular disease with neuroinflammation and brain disorders. J. Cereb. Blood Flow Metab. 36, 1668–1685. 10.1177/0271678x16662043 27486046 PMC5076791

[B29] Li C. M. Campbell S. J. Kumararatne D. S. Bellamy R. Ruwende C. McAdam K. P. W. J.. (2002). Association of a polymorphism in the P2X_7_ gene with tuberculosis in a gambian population. J. Infect. Dis. 186, 1458–1462. 10.1086/344351 12404161

[B30] Lindenau J. D. Salzano F. M. Guimarães L. S. P. Callegari-Jacques S. M. Hurtado A. M. Hill K. R.. (2013). Distribution patterns of variability for 18 immune system genes in Amerindians—relationship with history and epidemiology. Tissue Antigens 82, 177–185. 10.1111/tan.12183 24032724

[B31] Liu H. Han X. Li Y. Zou H. Xie A. (2013). Association of P2X7 receptor gene polymorphisms with sporadic Parkinson’s disease in a Han Chinese population. Neurosci. Lett. 546, 42–45. 10.1016/j.neulet.2013.04.049 23648388

[B32] Mokrousov I. Sapozhnikova N. Narvskaya O. (2008). Mycobacterium tuberculosis co-existence with humans: making an imprint on the macrophage P2X_7_ receptor gene? J. Med. Microbiol. 57, 581–584. 10.1099/jmm.0.47455-0 18436590

[B33] Najjar S. Pearlman D. M. Alper K. Najjar A. Devinsky O. (2013). Neuroinflammation and psychiatric illness. J. Neuroinflammation 10:43. 10.1186/1742-2094-10-43 23547920 PMC3626880

[B34] Novak I. Solini A. (2018). P2X receptor-ion channels in the inflammatory response in adipose tissue and pancreas—potential triggers in onset of type 2 diabetes? Curr. Opin. Immunol. 52, 1–7. 10.1016/j.coi.2018.02.002 29522971

[B35] Nückel H. Frey U. H. Dürig J. Dührsen U. Siffert W. (2004). 1513A/C polymorphism in the P2X7 receptor gene in chronic lymphocytic leukemia: absence of correlation with clinical outcome. Eur. J. Haematol. 72, 259–263. 10.1111/j.0902-4441.2003.00210.x 15089763

[B36] Ohlendorff S. D. Tofteng C. L. Jensen J. E. B. Petersen S. Civitelli R. Fenger M. (2007). Erratum: single nucleotide polymorphisms in the P2X7 gene are associated to fracture risk and to effect of estrogen treatment (Pharmacogenetics and Genomics (2007) 17, 7,(555–567)). Pharmacogenet. Genomics 17:787 10.1097/01.fpc.0000239983.92335.04 17558311

[B37] Özdemir F. A. Erol D. Konar V. Yüce H. KaraŞenli E. Bulut F.. (2014). Lack of association of 1513 A/C polymorphism in P2X_7_ gene with susceptibility to pulmonary and extrapulmonary tuberculosis. Tuberk. Toraks 62, 7–11. 10.5578/tt.4740 24814072

[B39] Sambasivan V. Murthy K. J. R. Reddy R. Vijayalakshimi V. Hasan Q. (2010). P2X7 gene polymorphisms and risk assessment for pulmonary tuberculosis in Asian Indians. Dis. Markers 28, 43–48. 10.3233/DMA-2010-0682 20164546 PMC3833339

[B40] Sanz J. M. Falzoni S. Rizzo R. Cipollone F. Zuliani G. Di Virgilio F. (2014). Possible protective role of the 489 >T P2X7R polymorphism in Alzheimer’s disease. Exp. Gerontol. 60, 117–119. 10.1016/j.exger.2014.10.009 25456845 PMC4266448

[B41] Savio L. E. B. de Andrade Mello P. da Silva C. G. Coutinho-Silva R. (2018). The P2X7 receptor in inflammatory diseases: angel or demon? Front. Pharmacol. 9:52. 10.3389/fphar.2018.00052 29467654 PMC5808178

[B42] Sellick G. S. Rudd M. Eve P. Allinson R. Matutes E. Catovsky D.. (2004). The P2X7 receptor gene A1513C polymorphism does not contribute to risk of familial or sporadic chronic lymphocytic leukemia. Cancer Epidemiol. Biomarkers Prev. 13, 1065–1067. 15184265

[B43] Shamsi M. Zolfaghari M. R. Farnia P. (2016). Association of IFN-γ and P2X7 receptor gene polymorphisms in susceptibility to tuberculosis among Iranian patients. Acta Microbiol. Immunol. Hung. 63, 93–101. 10.1556/030.63.2016.1.7 27020872

[B44] Sharma S. Kumar V. Khosla R. Kajal N. Sarin B. Sehajpal P. K. (2010). Association of P2X7 receptor +1513 (A→C) polymorphism with tuberculosis in a Punjabi population. Int. J. Tuberc. Lung Dis. 14, 1159–1163. 20819262

[B45] Singla N. Gupta D. Joshi A. Batra N. Singh J. (2012). Genetic polymorphisms in the *P2X7* gene and its association with susceptibility to tuberculosis. Int. J. Tuberc. Lung Dis. 16, 224–229. 10.5588/ijtld.11.0076 22137490

[B46] Sluyter R. Stokes L. (2011). Significance of P2X7 receptor variants to human health and disease. Recent Pat. DNA Gene Seq. 5, 41–54. 10.2174/187221511794839219 21303345

[B47] Somuk B. T. Koc S. Ates O. Göktas G. Soyalic H. Uysal I. O.. (2016). MBL, P2X7, and SLC11A1 gene polymorphisms in patients with oropharyngeal tularemia. Acta Otolaryngol. 136, 1168–1172. 10.1080/00016489.2016.1186835 27223255

[B48] Souza de Lima D. Ogusku M. M. Sadahiro A. Pontillo A. (2016). Inflammasome genetics contributes to the development and control of active pulmonary tuberculosis. Infect. Genet. Evol. 41, 240–244. 10.1016/j.meegid.2016.04.015 27101784

[B49] Sperlágh B. Illes P. (2014). P2X7 receptor: an emerging target in central nervous system diseases. Trends Pharmacol. Sci. 35, 537–547. 10.1016/j.tips.2014.08.002 25223574

[B100] Starczynski J. Pepper C. Pratt G. Hooper L. Thomas A. Hoy T.. (2003). The P2X7 receptor gene polymorphism 1513 A>C has no effect on clinical prognostic markers, in vitro sensitivity to fludarabine, Bcl-2 family protein expression or survival in B-cell chronic lymphocytic leukaemia. Br. J. Haematol. 123, 66–71. 10.1046/j.1365-2141.2003.04563.x 14510944

[B50] Tao J. H. Zhang Y. Li X. P. (2013). P2X7R: a potential key regulator of acute gouty arthritis. Semin. Arthritis Rheum. 43, 376–380. 10.1016/j.semarthrit.2013.04.007 23786870

[B51] Taype C. A. Shamsuzzaman S. Accinelli R. A. Espinoza J. R. Shaw M. A. (2010). Genetic susceptibility to different clinical forms of tuberculosis in the Peruvian population. Infect. Genet. Evol. 10, 495–504. 10.1016/j.meegid.2010.02.011 20188863

[B52] Tekin D. Kayaalti Z. Dalgic N. Cakir E. Soylemezoglu T. Isin Kutlubay B.. (2010). Polymorphism in the P2X7 gene increases susceptibility to extrapulmonary tuberculosis in Turkish children. Pediatr. Infect. Dis. J. 29, 779–782. 10.1097/inf.0b013e3181d9932e 20661107

[B53] Thunberg U. Tobin G. Johnson A. Söderberg O. Padyukov L. Hultdin M.. (2002). Polymorphism in the *P2X7* receptor gene and survival in chronic lymphocytic leukaemia. Lancet 360, 1935–1939. 10.1016/S0140-6736(02)11917-9 12493261

[B54] Vezzani A. French J. Bartfai T. Baram T. Z. (2011). The role of inflammation in epilepsy. Nat. Rev. Neurol. 7, 31–40. 10.1038/nrneurol.2010.178 21135885 PMC3378051

[B55] Wesselius A. Bours M. J. L. Arts I. C. W. Theunisz E. H. E. Geusens P. Dagnelie P. C. (2012). The P2X_7_ loss-of-function Glu496Ala polymorphism affects *ex vivo* cytokine release and protects against the cytotoxic effects of high ATP-levels. BMC Immunol. 13:64. 10.1186/1471-2172-13-64 23210974 PMC3526505

[B56] Wu J. Lu L. Zhang L. Ding Y. Wu F. Zuo W.. (2015). Single nucleotide polymorphisms in *P2X7* gene are associated with serum immunoglobulin g responses to mycobacterium tuberculosis in tuberculosis patients. Dis. Markers 2015:671272. 10.1155/2015/671272 26798189 PMC4698936

[B57] Xiao J. Sun L. Jiao W. Li Z. Zhao S. Li H.. (2009). Lack of association between polymorphisms in the P2X_7_ gene and tuberculosis in a Chinese Han population. FEMS Immunol. Med. Microbiol. 55, 107–111. 10.1111/j.1574-695X.2008.00508.x 19076224

[B58] Yeager M. Orr N. Hayes R. B. Jacobs K. B. Kraft P. Wacholder S.. (2007). Genome-wide association study of prostate cancer identifies a second risk locus at 8q24. Nat. Genet. 39, 645–649. 10.1038/ng2022 17401363

[B59] Zhang L. Y. Ibbotson R. E. Orchard J. A. Gardiner A. C. Seear R. V. Chase A. J.. (2003). P2X7 polymorphism and chronic lymphocytic leukaemia: lack of correlation with incidence, survival and abnormalities of chromosome 12. Leukemia 17, 2097–2100. 10.1038/sj.leu.2403125 12931211

